# Clinical implementation and error sensitivity of a 3D quality assurance protocol for prostate and thoracic IMRT

**DOI:** 10.1120/jacmp.v16i5.5392

**Published:** 2015-09-08

**Authors:** Gueorgui Gueorguiev, Christopher Cotter, Julie Catherine Turcotte, Bruce Crawford, Gregory Sharp, Mufeed Mah'D

**Affiliations:** ^1^ Biomedical Engineering and Biotechnology Program University of Massachusetts – Lowel Lowell MA; ^2^ Department of Radiation Oncology Massachusetts General Hospital Boston MA USA

**Keywords:** 3D quality assurance, IMRT, COMPASS

## Abstract

This work aims at three goals: first, to define a set of statistical parameters and plan structures for a 3D pretreatment thoracic and prostate intensity‐modulated radiation therapy (IMRT) quality assurance (QA) protocol; secondly, to test if the 3D QA protocol is able to detect certain clinical errors; and third, to compare the 3D QA method with QA performed with single ion chamber and 2D gamma test in detecting those errors. The 3D QA protocol measurements were performed on 13 prostate and 25 thoracic IMRT patients using IBA's COMPASS system. For each treatment planning structure included in the protocol, the following statistical parameters were evaluated: average absolute dose difference (AADD), percent structure volume with absolute dose difference greater than 6% (ADD6), and 3D gamma test. To test the 3D QA protocol error sensitivity, two prostate and two thoracic step‐and‐shoot IMRT patients were investigated. Errors introduced to each of the treatment plans included energy switched from 6 MV to 10 MV, multileaf collimator (MLC) leaf errors, linac jaws errors, monitor unit (MU) errors, MLC and gantry angle errors, and detector shift errors. QA was performed on each plan using a single ion chamber and 2D array of ion chambers for 2D and 3D QA. Based on the measurements performed, we established a uniform set of tolerance levels to determine if QA passes for each IMRT treatment plan structure: maximum allowed AADD is 6%; maximum 4% of any structure volume can be with ADD6 greater than 6%, and maximum 4% of any structure volume may fail 3D gamma test with test parameters 3%/3 mm DTA. Out of the three QA methods tested the single ion chamber performed the worst by detecting 4 out of 18 introduced errors, 2D QA detected 11 out of 18 errors, and 3D QA detected 14 out of 18 errors.

PACS number: 87.56.Fc

## I. INTRODUCTION

The increased complexity of treatment planning software and treatment delivery methods poses the risk of delivering erroneous dose to the patient during radiation treatment. This, combined with the risk of human or technical error, leads to the need of a more thorough process of performing quality assurance (QA). According to FDA adverse report from 03/14/2005,^(1)^ a patient underwent three fractions of the treatment with open field, potentially receiving total of 39 Gy dose in the head and neck region. Yeung et al.^(2)^ investigated 13,385 patients treated in a 10‐year period with 624 errors detected, concluding that human and technical errors will occur, but a proper QA procedure will reduce them significantly.

This work aims at three goals: to define a set of statistical parameters and plan structures for a 3D pretreatment thoracic and prostate intensity‐modulated radiation therapy (IMRT) quality assurance protocol; to test if the protocol is able to detect common and uncommon clinical errors; and to compare 3D QA method with traditional QA performed with either a single ion chamber or 2D gamma test.

Two of the most common IMRT QA measurement procedures are ion chamber point measurements and 2D dose measurements. Although there are certain advantages of both methods, there are commonly acknowledged drawbacks. A drawback for point measurement is its unreliability when the tumor size is similar to the chamber's active volume or the chamber is placed in high‐dose gradient areas.^(3)^ In addition, a single ion chamber measurement is a poor surrogate for analysis of a complex type of treatment such as IMRT. Another popular tool for performing IMRT QA is 2D measurement involving either film or a commercially available array of ion chambers. Once the measurement is acquired, a gamma analysis can be performed.^(4)^ One of the most commonly accepted analysis criteria is 3% dose difference and 3 mm distance to agreement (DTA). One disadvantage of this method for performing IMRT QA is weak‐to‐moderate correlation between 2D gamma analysis passing rates and clinically relevant errors. Nelms et al.^(5)^ introduced four types of errors and found lack of correlation between 2D gamma analysis passing rates and dose errors introduced to anatomic regions of interest. Budgell et al.^(6)^ concluded that, when 2D gamma analysis is performed using 3% dose difference and 3 mm DTA, the result and the error detectability is strongly dependent on the plane chosen for measurement acquisition, and no correlation could be found between the levels of errors in different verification planes. Both research teams suggest that moving from 2D to 3D gamma analysis might solve those issues. The most popular methods of performing 2D gamma analysis use either 2D ion chamber or diode arrays. Hussein et al.^(7)^ investigated five commercial QA systems, including 2D ion chamber array PTW 2D‐array and 2D diode arrays ScandiDos ${\text{Delta}}^{4}$ and Sun Nuclear ArcCHECK. The author concluded that good statistical agreement exists between predicted gamma based on virtual measurement relative to independent prediction. The general direction in which QA is moving is the implementation of patient specific 3D QA protocol. There are various products on the market that are able to perform 3D dose reconstruction based on 2D measurement, including Standard Imaging Dosimetry Check, ScandiDos ${\text{Delta}}^{4}$, Sun Nuclear 3DVH, IBA COMPASS, and PTW OCTAVIUS 4D. Considerable research has been done in development of 3D QA procedures. Van Elmpt et al.^(8)^ concluded that EPID dosimetry combined with 3D dose reconstruction is a useful procedure for patient‐specific QA of complex treatments. Wu et al.^(9)^ concluded that 3D gamma analysis based on EPID dose back‐projection may provide a feasible tool for IMRT and VMAT pretreatment plan QA. Fan et al.^(10)^ concluded that the 3D diode device ArcCHECK with a 90% pass rate using gamma criteria of 3%/3 mm distance to agreement (DTA) and is suitable for patient QA. Visser et al.^(11)^ introduced hybrid QA incorporating both model‐based and measurement‐based QA. Korevaar et al.^(12)^ compared film and COMPASS measurements for both test and 24 head and neck IMRT plans. Bogulla et al.^(13)^ validated the dosimetric performance of COMPASS for VMAT plans. In another study they also evaluated two detector types, MatriXX detector and transmission detector, for prostate IMRT.^(14)^

Previous research on error detectability during QA has also been done, but was restricted mainly to theoretical work or QA using 2D gamma analysis. Carver et al.^(15)^ stated that different statistical tests detect different errors and that some clinical errors can be detected by gamma evaluation, while others can be detected solely by investigating mean dose.

Rivest et al.^(16)^ concluded that 3D gamma analysis can successfully identify MLC leaf pair shifting errors during prostate and head and neck IMRT. Childress et al.^(17)^ performed QA using 2D gamma analysis and normalized agreement test on nine prostate and seven paranasal sinus cases. Both authors concluded QA using 2D gamma analysis is superior to a single ion chamber measurement. García‐Vicente et al.^(18)^ concluded that 2D gamma analysis QA with 3%/2 mm DTA parameters and measurement acquired with Sun Nuclear ArcCHECK is sufficient to detect random and systematic variation of gantry angle and systematic errors in multileaf collimator (MLC) gap width on three dynamic IMRT head and neck and prostate cases. Godart et al.^(19)^ investigated IBA COMPASS ability to detect MLC positioning errors up to 10 mm and concluded that the correction kernel method used by the COMPASS system is adequate to perform QA of IMRT treatment plans with a regular MLC, despite local inaccuracies in the dose reconstruction.

We feel that more clinical research work needs to be done, in particular by developing rigorous 3D QA protocols utilizing multiple statistical parameters applied to any treatment plan structure of interest. This, we believe, will improve the QA process and the error detectability during QA.

## II. Materials and Methods

### A.1 Quality assurance with single ion chamber and I'MRT MatriXX software

For single ion chamber point IMRT QA, each patient's clinically approved plan was recalculated on an epoxy resin Solid Water phantom with dimensions 30×30×22 cm containing an ion chamber at its center. The TPS calculates the expected dose in the chamber's active volume. Afterwards, an ion chamber was used to measure absolute dose on the treatment machine. The point for ion chamber measurement that we typically select is one in high‐dose, low‐dose gradient area, which in the majority of cases is at the isocenter. In this work we performed ion chamber QA with two chamber locations, one at isocenter and the other 1.5 cm off isocenter in inferior direction. The later was used to show that most likely errors directly above the ion chamber in the beam line will be detected, and those away will most likely be not (in our case away was 1.5cm). Our ion chamber QA protocol includes two threshold values, 4% and 6%. QA is a pass if computed and measured dose difference is within 4% and a conditional pass if the difference is within 6%. Conditional pass means that the patient will start treatment as scheduled, but an ion chamber QA will be performed again, this time using another point for QA.

Additionally, measurement for all patients was acquired with IBA's I'MRT MatriXX system (IBA Dosimetry, Schwarzenbruck, Germany) and a 2D gamma test was performed. Gamma test parameters were: 3%/3 mm DTA and 10% maximum dose cutoff. When a patient treatment plan was exported from RayStation (RaySearch Laboratories, Stockholm, Sweden) to be used in I'mRT MatriXX software for gamma analysis, we set the gantry and collimator angles at 0°. During measurement acquisition we use 5 cm solid water on top of the MatriXX detector. After acquisition, 2D gamma analysis is performed on the composite of all fields. In our clinic, gamma test with parameters 3%/3 mm DTA to pass QA at least 90% of the data points must pass the test. If between 85% and 90% pass, we consider it a conditional pass and they are evaluated case‐by‐case. Detailed 2D gamma analysis has been described previously.^(4)^ The system's detector consists of a 2D array of 1020 vented ion chambers in a 32×32 grid.

### A.2 IBA's COMPASS system

The tool we investigated for 3D IMRT QA was IBA's COMPASS system that consists of a similar 2D array of 1020 vented parallel ion chambers detector mounted on the linear accelerator gantry. The ion chambers are arranged in a 32×32 grid, except for the four corner positions, have $0.08\;{\text{cm}}^{3}$ active volume, and are spaced at 7.62 mm center to center of chambers. The detector has 3 mm absorber material placed above the chambers, is connected to a laptop using an Ethernet cable, and to an angle sensor which records the gantry angle at which measurements are taken. It is mounted on the accelerator gantry with source‐to‐surface distance of 76.2 cm, and uses an additional 5 cm of solid water buildup. The detector is placed perpendicular to the X‐ray fluence, and rotates with the gantry and collimator while measuring the X‐ray fluence. The acquired fluence data are used to compute the dose in the patient CT using a collapsed cone 3D convolution algorithm. Details of the COMPASS system have been previously published.^13^, ^20^

### A.3 Patient and treatment plan selection for clinical implementation of a 3D QA protocol for thoracic and prostate IMRT

In this work, we chose as diverse initial IMRT plans as possible from both clinics. Thirteen prostate and 25 thoracic IMRT patients were selected to commission 3D IMRT system. Prostate cases were measured four times on three different beam‐matched Elekta Infinity accelerators (Elekta, Stockholm, Sweden), with measurements on two different days for one of the accelerators. This was done to confirm measurement consistency between all Elekta Infinity accelerators in our institution and between different days. Thoracic cases were measured once. All patient plans have passed single ion chamber QA and 2D gamma analysis (Table 1) as a part of our standard QA routine. All patients were planned on RaySearch Raystation (RaySearch Laboratories) treatment planning system (TPS) with step‐and‐shoot IMRT. Both initial and boost treatments were investigated. Prostate cases were seven‐ to nine‐field IMRT plans including prostate, seminal vesicles, prostate bed, and lymph nodes as treatment sites. Initial prescription dose was 42–45 Gy and the dose for the boost was 18–34 Gy, depending on the treatment protocol and disease stage. Thoracic cases were five‐ to seven‐field IMRT plans including lung, esophagus, and lymph nodes as treatment sites. Initial treatment prescription doses were 40–60 Gy, and boost treatment prescription doses were 10–30 Gy.

**Table 1:** Statistical analysis on single ion chamber and 2D gamma test QA results. Single ion chamber results represent the TPS computed vs. measured dose difference in percent. For ion chamber QA pass/fail criterion is 6%, 2D gamma criterion (3%/3 mm DTA) is 85%

	*13 Prostate Cases*		*25 Thoracic Cases*	
*Statistical Parameter*	*2D gamma pass rates (%)*	*single ion chamber*	*2D gamma test pass rates (%)*	*single ion chamber*
Minimum	91.89	−2.76	87.40	−1.70
Maximum	99.13	1.36	98.76	4.28
SD	2.42	1.29	3.04	1.51
Median	94.00	−0.41	94.84	1.08
Range	7.24	4.12	11.36	5.98
Average	94.61	−0.66	94.96	1.41

### A.4 Treatment plan structure selection

Based on IMRT planning protocols implemented in our institution, we selected the following plan structures to be included in the 3D IMRT QA protocol. Prostate cases: prostate, seminal vesicles, femoral heads, whole rectum, anterior and posterior rectum or rectum wall, and bladder. Thoracic cases: target treatment site, healthy lungs, spinal cord, heart, ventricles, atria, and esophagus.

### A.5 Statistical parameters included in QA protocol

Statistical parameters that were included in the 3D IMRT protocol for each plan structure are: AADD, ADD6, and 3D gamma analysis. The first statistical parameter is average absolute dose difference (AADD) which compares for each structure average dose predicted by the TPS and the one which is measured. The average dose is computed by summing the dose values for all voxels falling into given structure and dividing by the number of voxels. The average dose difference between both doses is computed using

(1) $$Average\;dose\;[\%]=\frac{TPS\;avg.dose-COMPASS\;Measured\;avg.dose}{TPS\;avg.dose}*100$$

The second parameter is structure volume with an absolute dose difference greater than 6% (ADD6). The system evaluates two dose matrices, TPS predicted and measured. Both consist of the same number of voxels and structures. COMPASS will compute for any plan structure the absolute dose difference deposited in a voxel with the same location in both matrixes. If the absolute dose difference is greater than 6%, this voxel pair fails QA. The total percent of each structure volume that fails this statistical parameter is calculated and reported. We investigated three possible absolute dose difference thresholds, 1%, 3%, and 6%, and found the first two to be too restrictive, resulting in false QA failures in plans that pass traditional point and 2D measurements. In addition, comparison of RayStation TPS‐computed and COMPASS‐computed doses fail at these thresholds. The final parameter is 3D gamma analysis with computation done globally and test parameters: 3%/3 mm DTA and 10% maximum dose cutoff. The principle of 3D gamma analysis is described previously.^(21)^ We chose those analysis parameters after determining that gamma test parameters of 1%/1 mm DTA, 2%/2 mm DTA were too conservative, resulting in false QA failure results. Similarly to ADD6%, such conservative test parameters resulted in 3D gamma fail for plans that pass traditional point and 2D measurements. Between 30%–65% for most of investigated structures failed, even when RayStation TPS computed and COMPASS computed doses were evaluated.

### A.6 Clinical implementation of 3D IMRT QA protocol

After selecting treatment plan structures and statistical parameters applied to each structure, statistical parameter threshold values (Table 2) were determined based on measuring four times 13 prostate patients and once times 25 thoracic ones. After the threshold values were determined and the 3D QA protocol was implemented for the prostate clinic, an additional 50 prostate patients were measured. For those, only 3D QA was performed. Prostate results shown in this work include both the initial 13 and the additional 50 patients. The AADD, ADD6, and 3D gamma threshold values of 6%, 4%, and 4% were based on the maximum measured values from both clinics, respectively 4.4%, 3.8% and 3.67% (3, 4).

**Table 2:** Complete 3D QA protocol for prostate and thoracic IMRT

		*3D Statistical Parameter*		
*CLINIC*	*Plan Structure*	*AADD*	*ADD6*	*3D Gamma Analysis With Parameters* 3%/3 mm *DTA*
Thoracic	treatment volume, healthy lungs, spinal cord, heart, ventricles, atria, esophagus	For QA to pass: 6% is the maximum allowed TPS computed and COMPASS measured average dose difference	For QA to pass: maximum of 4% structure volume may have TPS computed and COMPASS measured absolute dose difference greater than 6%	For QA to pass: maximum 4% of any given structure volume may fail 3D gamma test
Prostate	prostate, seminal vesicles, femoral heads, whole rectum, anterior and posterior rectum, bladder			

**Table 3:** Statistical analysis on 3D IMRT QA parameters for 102 measurements of 63 prostate cases. Statistical parameters threshold values determining if QA passes for any plan structure are: 6% for AADD, 4% for ADD6, and 4% for 3D gamma test

	*Anterior Rectum*	*Bladder*	*Left Femoral Head*	*Posterior Rectum*	*Prostate And Fossa*	*Right Femoral Head*	*Seminal Vesicles*	*Rectum*	*Statistical Parameter*
Minimum	0.11	0.00	0.00	1.00	0.00	0.00	0.00	0.60	AADD (%)
Maximum	4.30	4.40	4.16	4.10	3.53	4.13	3.77	4.00	
SD	1.00	0.96	1.11	0.73	0.85	1.14	0.94	0.80	
Median	2.70	1.40	1.65	2.90	0.82	1.70	1.31	2.70	
Range	4.19	4.40	4.16	3.10	3.53	4.13	3.77	3.40	
Average	2.54	1.53	1.78	2.84	1.02	1.70	1.37	2.63	
Minimum	0.00	0.00	0.00	0.00	0.00	0.00	0.00	0.00	ADD6 (%)
Maximum	3.80	3.49	1.30	2.16	1.79	3.22	1.77	1.35	
SD	1.06	0.76	0.18	0.36	0.20	0.49	0.32	0.30	
Median	0.41	0.10	0.00	0.00	0.00	0.00	0.00	0.07	
Range	3.80	3.49	1.30	2.16	1.79	3.22	1.77	1.35	
Average	0.89	0.49	0.04	0.11	0.03	0.19	0.11	0.19	
Minimum	0.00	0.00	0.00	0.00	0.00	0.00	0.00	0.00	Structure volume that fails 3D gamma (%)
Maximum	3.23	2.84	2.82	2.03	3.37	3.41	3.67	0.62	
SD	0.91	0.41	0.47	0.29	0.79	0.76	0.70	0.13	
Median	0.00	0.00	0.00	0.00	0.00	0.00	0.00	0.00	
Range	3.23	2.84	2.82	2.03	3.37	3.41	3.67	0.62	
Average	0.55	0.15	0.20	0.09	0.37	0.40	0.26	0.05	

**Table 4:** Statistical analysis on 3D IMRT QA parameters for 25 thoracic measurements. Statistical parameters threshold values determining if QA passes for any plan structure are: 6% for AADD, 4% for ADD6, and 4% for 3D gamma test

	*Atria*	*Esophagus*	*Heart*	*Liver*	*Left Lung*	*Right Lung*	*Spinal Cord*	*Target*	*Ventricles*	*Statistical Parameter*
Minimum	0.20	0.10	0.40	0.20	0.10	0.10	0.10	0.10	0.60	AADD (%)
Maximum	2.70	3.50	3.80	4.10	4.20	4.20	4.10	2.60	4.10	
SD	0.65	1.04	1.49	1.30	1.04	0.94	1.14	0.72	1.02	
Median	1.45	1.55	1.80	1.90	1.80	1.80	1.80	1.10	1.80	
Range	2.50	3.40	3.40	3.90	4.10	4.10	4.00	2.50	3.50	
Average	1.45	1.71	2.00	1.83	1.81	1.81	1.80	1.13	2.06	
Minimum	0.00	0.00	0.00	0.00	0.00	0.00	0.00	0.00	0.00	ADD6 (%)
Maximum	0.61	0.67	0.81	0.01	0.08	0.07	0.00	0.07	0.10	
SD	0.17	0.19	0.25	0.00	0.02	0.02	0.00	0.02	0.03	
Median	0.00	0.00	0.01	0.00	0.00	0.00	0.00	0.00	0.00	
Range	0.61	0.67	0.81	0.01	0.08	0.07	0.00	0.07	0.10	
Average	0.05	0.11	0.10	0.00	0.01	0.01	0.00	0.01	0.02	
Minimum	0.00	0.00	0.00	0.00	0.00	0.00	0.00	0.00	0.00	Structure volume that fails 3D gamma (%)
Maximum	0.01	1.71	0.23	0.41	0.95	0.31	0.64	2.52	0.08	
SD	0.00	0.41	0.07	0.12	0.24	0.07	0.14	0.90	0.02	
Median	0.00	0.00	0.00	0.00	0.00	0.00	0.00	0.03	0.00	
Range	0.01	1.71	0.23	0.41	0.95	0.31	0.64	2.52	0.08	
Average	0.00	0.16	0.03	0.04	0.09	0.02	0.04	0.67	0.00	

### B.1 Patient and treatment plan selection for error sensitivity testing of a 3D QA protocol for thoracic and prostate IMRT

Two prostate and two thoracic patients treated with step‐and‐shoot IMRT were selected for error sensitivity testing. Both prostate plans were with seven fields, included prostate and seminal vesicles as treatment sites, and had a prescribed dose of 45 Gy. The first thoracic plan was with five fields, treated a lesion in the left lung, and had a prescribed dose of 69.9 Gy. The second thoracic plan was with five fields and treated a lesion in the esophagus with prescription dose of 41.4 Gy. All patients were planned on RaySearch RayStation TPS and treated on an Elekta Infinity linear accelerator. Prostate plan structures analyzed were: prostate, seminal vesicles, femoral heads, whole rectum, anterior and posterior rectum, and bladder. Thoracic plan structures analyzed were: esophagus, treatment volume, left and right lung, and spinal cord.

### B.2 Introduced errors and clinical significance

To test if our selected tolerance levels can detect common clinical errors, we introduced a number of such errors into the treatment delivery. These included energy changed from 6 MV to 10 MV; linac jaws retracted to 15 cm by 15 cm from original smaller position; 1, 2, or 3 central (MLC) leaf pairs retracted behind the jaws; single central leaf inserted into or retracted from all segments of all treatment fields by 2–6 mm; monitor units (MU) increased or decreased for all fields by 1% and 3%; collimator angle changed by 5° and 10°; detector shifted by 5 mm to the left or right; and gantry treatment angle changed by 5° or 15°. The introduced errors have the following clinical impact, central MLC leafs, and MU change errors will affect mostly the target structures, retracting the jaws will affect primarily the organs at risk (OAR). For example, MLC leaves retracted caused clinically unacceptable dose elevation between 5%–50% for the bladder, between 25%–105% for the femoral heads, and between 2%–22% for the anterior rectum. When the linac jaws were retracted, the bladder and the femoral heads received on average 20% higher dose.

## III. RESULTS

### A. 3D IMRT QA parameter measurement results and QA pass tolerance levels

3, 4 show statistical analysis on 3D IMRT QA parameters for 102 prostate and for 25 thoracic measurements. From these measurement results we propose tolerance level for the first parameter that will determine if QA passes to be 6%, meaning 6% is the maximum allowed AADD. For both treatment sites investigated we propose tolerance level for the second parameter that will determine if QA passes to be 4%, meaning maximum of 4% structure volume may have ADD6. From the measurement results of the two treatment sites investigated we propose tolerance level for the final parameter, 3D gamma test that will determine if QA passes to be 4%, meaning maximum 4% of any given structure volume may fail 3D gamma test. Those tolerance levels comprise the 3D QA protocol as shown on Table 2

#### B.1 Single ion chamber QA results for errors introduced

Table 5 shows results from single ion chamber QA. Without introduced errors, results met the 6% QA pass threshold used in our institution. Gross errors, such as all MLC leaves retracted behind linac jaws and 1, 2, or 3 central leaf pairs retracted behind jaws, were detected as a failure by ion chamber QA for all four patients and for all ion chamber positions. When the ion chamber is 1.5 cm off the isocenter and a pair of central MLC leaves is retracted for both prostate and thoracic patients the error was not detected. The jaws retracted error was not detected because the chamber is positioned in the treatment field, while this error causes MLC interleaf leakage outside of the treatment field. Errors of a single central MLC leaf inserted into or retracted from the field were not being detected by this QA method due to their subtlety, with the one exception of the first prostate patient. MU change was not being detected by the ion chamber QA due to the pass/fail threshold of 6% used in our institution. 10 MV error was not detected. Collimator and gantry angle errors were not detected by ion chamber QA. Typically, the ion chamber is placed near the middle of the treatment field and, while performing QA in Solid Water phantom, changing the treatment plan gantry or MLC angle will result only in changing the photon path length through solid water material or in rotating the treatment field, both of which will not result in QA fail. Detector shifted by 5 mm errors were not detected, as well. When we select QA point in the treatment plan, we choose one that is not in a steep dose gradient areas. Therefore, shifting the detector by 5 mm is usually still in the same dose area and QA output will not change significantly.

**Table 5:** Complete QA results of the introduced errors. For ion chamber QA pass/fail criterion is 6%, 2D gamma criterion (3%/3 mm DTA) is 85%. 3D QA pass/fail criteria are 6% for AADD, 4% for ADD6, and 4% for 3D gamma test. 3D QA results present the final result, either pass or fail, after plan review. Green background indicated a plan that passed QA according our QA protocol, red background indicated a failed plan

	*Single Ion Chamber QA Results*					*2D QA Results*					*3D QA Results*			
*Error*	*Prostate Case #1*	*Prostate Case #1*	*Prostate Case #2*	*Thoracic Case #1*	*Thoracic Case* #*1*	*Thoracic Case #2*	*Prostate Case* #*1*	*Prostate Case #2*	*Thoracic Case* #*1*	*Thoracic Case #2*	*Prostate Case* #*1*	*Prostate Case #2*	*Thoracic Case* #*1*	*Thoracic Case #2*
*Ion Chamber Measurement Taken At*:	*Isocenter*	*1.5 cm off Isocenter*	*Isocenter*	*Isocenter*	*1.5 cm off Isocenter*	*Isocenter*	*Volume that passes 2D gamma test with parameters: 3% dose difference/3 mm DTA*							
	*TPS vs. Measured Absolute Dose Difference (%)*													
no error	1.19	0.79	−0.34	−0.3	1.32	1.75	92.93	93.16	98.71	93.6	Pass	Pass	Pass	Pass
10 MV	4.64		5.93	2.85		5.34	96.87	94.52	96.36	97.44	Pass	Pass	Pass	Pass
jaws retracted	2.8	**a**	2.96	3.08	**a**	15.71	39.22	60.67	27.59	47.85	Fail	Fail	Fail	Fail
MLC retracted behind jaws	32.37		28.77	26.24		72.77	9.62	3.7	1.22	9.08	Fail	Fail	Fail	Fail
1 central leaf pair behind jaws	7.77	0.69	12.6	10.16	3.16	17.31	69.9	65.04	56.85	89.11	Fail	Fail	Fail	Fail
2 central leaf pairs behind jaws	21.69	11.7	18.7	21.01	13.08	26.89	49.99	55.93	40.21	78.55	Fail	Fail	Fail	Fail
3 central leaf pairs behind jaws	24.91	11.88	23.69	25.4	32.48	48.43	35.62	47.46	28.92	70.2	Fail	Fail	Fail	Fail
single central leaf in	−7.04	0.31	−5.41	−6.5	2.65	−6.63	74.45	90.73	80.5	89.51	Fail	Fail	Pass	Pass
single central leaf out	3.94	0.02	5.86	2.27	2.24	4.94	80.93	84.31	89.02	81.31	Fail	Fail	Fail	Fail
−1% MU	−1.61		−3.29	−1.04		0.15	94.35	90.01	96.19	92.66	Pass	Pass	Pass	Pass
−3% MU	−3.37	**a**	−5.41	−3.18	**a**	−3.44	88.67	84.4	90.9	89.21	Fail	Fail	Fail	Fail
1% MU	2.53		0.38	0.9		3.75	96.14	92.75	98.53	92.76	Pass	Pass	Pass	Pass
3% MU	4.28		2.18	2.78		6.74	89.41	91.13	88.94	91.05	Fail	Fail	Fail	Fail
MLC rotated to +5%	−0.18	1.16	−1.19	1.3	1.47	−0.25		Error not detected due to QA setup nature						
MLC rotated to +10%	−0.27	1.79	−1.54	3.04	0.86	−4.24								
detector shifted 5 mm to the left	1.05	−0.11	−0.74	1.52	0.39	20.1	87.5	90.19	90.29	89.8	Fail	Fail	Fail	Fail
detector shifted 5 mm to the right	1.22	−0.46	−1.24	1.94	−0.57	−9.02	88.8	91.87	89.8	87.88	Fail	Fail	Fail	Fail
gantry off by 5 °	−0.08	0.96	−0.92	1.2	1.25	−0.25	Error not detected due to QA setup nature				Fail	Fail	Fail	Fail
gantry off by 15°	−1.7	−0.34	2.51	−0.11	−0.22	−0.64		Fail	Fail	Fail	F9

a An error that is independent of chamber position since it affects the whole treatment field.

#### B.2 2D gamma analysis QA results for errors introduced

Table 5 shows results for the 2D gamma analysis QA. Without introduced errors, results were within the 90th percentile pass criterion. The 10 MV error was not detected. All MLC leaf errors were detected with this QA method and QA failed, with the exception of the second prostate patient with single central leaf inserted into the field. MU change by 1% error was not detected, while MU change by 3% was. In our institution 2D QA is performed with gantry and collimator at 0° for all treatment fields and, therefore, collimator and gantry angle errors were undetectable. Detector shift error produced mixed results all near the 90% pass criterion.

#### B.3 3D QA results for errors introduced

5, 6, 7 show the 3D QA results. 3D QA results in Table 5 represent the final decision if a plan passes or fails based on all structures for that clinical site. 6, 7 present detailed 3D QA results for subtle errors. Without introduced errors, results for all four patients were within the pass threshold levels set in Table 2. Gross errors, such as MLC leaf pair errors or detector shift, were detected by our QA protocol. AADD with a threshold value of 6% was not sensitive to subtle errors such as MU change, single MLC leaf in the field, or 10 MV energy change. From the organs at risk (OAR) investigated, femoral heads, rectum, esophagus, and spinal cord appeared to be the planning structures most sensitive to the introduced errors. Table 6 shows AADD for the prostate patients, and Table 7 for the thoracic patients. Different results for single leaf inserted into the field and retracted from the field were due to the fact that the leaf could be retracted out of the field for all treatment plan MLC segments, but the same leaf cannot be always inserted into the field since for some of the segments it was already closed.

For all four patients, the second parameter in our QA protocol, ADD6 with threshold of a 4%, detected gross clinical errors such as MLC leaf pair errors, MLC leaves retracted errors, and detector shifted by 5 mm errors. For all four patients, subtle errors, such as MU or energy change, were not being detected by this QA parameter. Due to reasons mentioned above, results for a single MLC leaf inserted into or retracted from the field errors vary.

The results for the final parameter in our QA protocol, 3D gamma, indicate that this parameter with a threshold value of 4%, meaning 4% is the allowed structure volume to fail 3D gamma analysis, was the most sensitive out of the three included in the QA protocol. It detected all gross errors for all patients and was also sensitive to subtle errors such as single MLC leaf inserted into or retracted from the treatment field, and MU changed by 3%. This was especially valid for the target structures prostate, seminal vesicles and target.

**Table 6:** 3D QA results for subtle errors introduced to two prostate patients. Statistical parameters threshold values determining if QA passes for any plan structure are: 6% for AADD, 4% for ADD6, and 4% for 3D gamma test. Green background indicated a plan that passed QA according our QA protocol; red background indicated a failed plan

*Error*	*Error Free*			−3% MU			+3% MU			*Single Leaf In*	*Single Leaf Out*			*Jaws Retracted*		
			*volume that fails 3D*			*volume that fails 3D*			*volume that fails 3D*			*volume that fails 3D*			*volume that fails 3D*			*volume that fails 3D*
*Stat. Parameter*	*AADD*	*ADD6*	*gamma*	*AADD*	*ADD6*	*gamma*	*AADD*	*ADD6*	*gamma*	*AADD*	*ADD6*	*gamma*	*AADD*	*ADD6*	*gamma*	*AADD*	*ADD6*	*gamma*
patient 1																	
Prostate	1.00	0.00	0.00	3.40	2.10	57.33	3.40	1.80	66.16	4.60	35.54	31.09	4.60	35.54	39.02	1.90	5.01	0.80
Anterior rectum	0.30	0.82	0.00	6.30	3.80	4.10	5.10	3.02	4.80	2.60	7.06	2.09	2.60	7.06	1.09	12.00	31.15	37.49
Posterior rectum	1.00	0.00	0.00	6.90	0.00	0.00	6.20	0.00	0.00	4.20	0.15	0.39	4.20	0.15	0.48	12.50	15.00	10.81
Bladder	5.30	0.50	0.00	1.30	2.70	1.21	5.80	3.45	1.80	1.20	0.87	0.13	1.20	0.87	0.19	20.80	28.77	35.79
Seminal vesicles	0.80	0.00	0.00	3.30	2.50	53.06	3.50	3.00	65.32	0.00	5.00	19.21	0.00	9.20	20.38	2.50	0.00	15.45
R femoral head	2.50	0.00	0.00	5.40	0.00	0.00	7.20	0.02	0.00	20.20	20.78	18.00	20.20	20.78	24.18	19.80	4.48	14.26
L femoral head	1.90	0.00	0.00	2.90	0.00	0.00	6.20	0.00	0.01	12.70	9.94	11.05	12.70	9.94	12.28	18.20	2.33	8.89
patient 2																		
Prostate	1.00	0.00	0.00	2.22	3.40	41.00	4.26	3.20	74.53	0.91	12.33	23.63	3.45	22.99	26.28	5.34	7.05	50.09
Anterior rectum	0.35	0.00	0.00	4.82	2.94	4.66	2.50	2.28	4.78	4.18	11.69	5.60	2.93	14.79	9.67	8.37	22.70	18.77
Posterior rectum	0.69	0.00	0.00	4.65	0.00	0.00	2.80	0.00	0.00	4.95	1.82	11.41	8.32	3.05	13.58	13.86	6.06	17.95
Bladder	4.90	1.82	0.08	0.46	3.23	2.80	6.03	3.52	1.61	0.90	6.34	5.31	5.46	7.37	3.77	17.37	24.42	26.10
Seminal vesicles	0.83	0.00	0.41	2.44	3.20	43.09	3.88	3.41	65.97	0.58	18.68	25.82	3.24	21.19	28.12	5.37		40.40
R femoral head	2.01	0.00	0.00	3.28	0.34	1.34	3.62	0.00	0.16	2.79	11.60	11.79	10.87	13.31	14.90	11.38	0.65	18.35
L femoral head	1.11	0.00	0.00	2.75	0.00	0.18	3.35	0.00	0.00	4.94	5.17	6.37	7.33	6.41	8.55	12.30	11.01	14.98

**Table 7:** 3D QA results for subtle errors introduced to two thoracic patients. Statistical parameters threshold values determining if QA passes for any plan structure are: 6% for AADD, 4% for ADD6, and 4% for 3D gamma test. Green background indicated a plan that passed QA according our QA protocol; red background indicated a failed plan

*Error*	*Error Free*			−3% MU			+3% MU			*Single Leaf In*			*Single Leaf Out*			*Jaws Retracted*		
			*volume that fails 3D*			*volume that fails 3D*			*volume that fails 3D*			*volume that fails 3D*			*volume that fails 3D*			*volume that fails 3D*
*Stat. Parameter*	*AADD*	*ADD6*	*gamma*	*AADD*	*ADD6*	*gamma*	*AADD*	*ADD6*	*gamma*	*AADD*	*ADD6*	*gamma*	*AADD*	*ADD6*	*gamma*	*AADD*	*ADD6*	*gamma*
patient 1																		
Esophagus	0.30	0.00	0.00	3.60	0.00	0.00	4.50	0.00	0.00	2.40	0.24	5.00	3.60	6.43	8.44	35.70	36.61	57.08
Target	0.00	0.00	0.00	3.00	0.00	15.09	2.90	0.00	22.49	10.30	30.50	6.10	13.60	40.54	8.05	2.50	12.25	8.53
Lt Lung	1.40	0.00	0.00	4.70	0.53	0.36	4.20	0.00	1.10	2.79	0.19	0.00	3.05	0.57	0.10	36.30	47.48	6.12
Rt Lung	1.70	0.00	0.00	6.70	0.00	0.00	8.20	0.00	0.05	8.23	0.73	2.10	9.80	0.94	3.76	52.30	5.28	10.93
Spinal Cord	0.70	0.00	0.00	2.60	0.00	0.20	3.40	0.00	0.67	0.89	4.88	0.00	0.00	4.51	1.11	46.90	59.86	34.14
patient 2																		
Esophagus	1.30	0.00	0.00	4.20	0.00	14.03	4.68	0.00	15.19	4.00	3.73	5.03	4.53	5.35	6.37	28.66	15.34	57.76
Target	0.48	0.00	0.00	10.36	0.00	26.65	9.65	0.00	40.27	5.06	5.66	6.65	6.74	6.89	11.45	0.00	4.14	3.87
Lt Lung	2.91	0.00	0.00	8.66	0.00	0.00	11.38	0.00	0.00	0.33	4.36	0.00	0.48	5.84	0.01	37.07	42.46	65.80
Rt Lung	0.60	0.00	0.00	3.30	0.00	1.37	3.45	0.00	1.23	0.02	3.72	1.37	0.05	0.53	3.55	20.82	8.27	17.80
Spinal Cord	0.69	0.00	0.00	4.20	0.00	0.00	4.97	0.00	0.30	1.50	3.20	0.27	2.52	4.61	0.31	45.03	29.54	46.46

## IV. DISCUSSION

The increased complexity of radiation therapy delivery, paired with the danger of human or hardware errors, make the process of quality assurance a pressing matter. The process of QA aims at two goals, to verify the patient's radiation treatment plan and to minimize the occurrence of delivery errors. As previously mentioned, performing IMRT QA with single ion chamber and 2D array of ion chambers used to acquire measurement for gamma analysis have certain disadvantages. In this work we aimed at addressing those disadvantages and improving the QA process by introducing certain novel features available only through 3D QA. One such feature is the ability to utilize three diverse statistical parameters on any treatment plan structure (Table 2). We decided to use three statistical parameters — gamma analysis, AADD, and ADD6 — since either parameter was more sensitive to certain errors only. For instance, AADD was more sensitive to global errors affecting the whole treatment field such as MU changes, while ADD6 was more sensitive to local errors affecting only part of the treatment field such as single MLC leaf errors. This can be seen in 6, 7

We also tested our 3D QA protocol sensitivity to some clinical errors. Two of which were not detected by any of the QA methods tested were beam energy changed to 10 MV and MU change by 1%. One percent MU change was too subtle of an error. For our Elekta accelerators, the dose difference between 6 and 10 MV at depths of 5 or 10 cm of solid water (5 cm used with 3D and 2D gamma measurement QA, and 10 cm used with single ion chamber QA) varies by field size between 0% and 4%. The pass/fail thresholds for the three QA methods investigated were not sensitive enough to detect the 10 MV error for the clinical plans tested. The worst performing out of the three QA methods was the ion chamber measurement. It conclusively detected 4 out of the 18 introduced errors. In this QA method, error detectability was also dependent on the error location. For instance, a single MLC leaf pair retracted from the field error could not be detected with ion chamber placed 1.5cm off the isocenter. MLC and gantry angle errors passed ion chamber QA. 2D gamma analysis was not performed on those errors due to setup used in our institution, where all beams are delivered at gantry and MLC at 0°. Similarly, during 3D QA the detector rotates with the MLC and collimator angle errors were not detected. 3D QA was sensitive to gantry position errors since it utilizes an angle sensor. The system will permit data acquisition at incorrect gantry angles, but will display warning regarding the mismatch during measurement analysis. 2D QA proved to outperform single ion chamber QA in detecting 11 out of the 18 introduced errors. 3D QA showed to be the most sensitive to the introduced errors by detecting 14 out of 18 errors. Out of the three statistical parameters used in our QA protocol, AADD was the least sensitive to the introduced errors, while 3D gamma analysis was the most sensitive.

**Figure 1:**
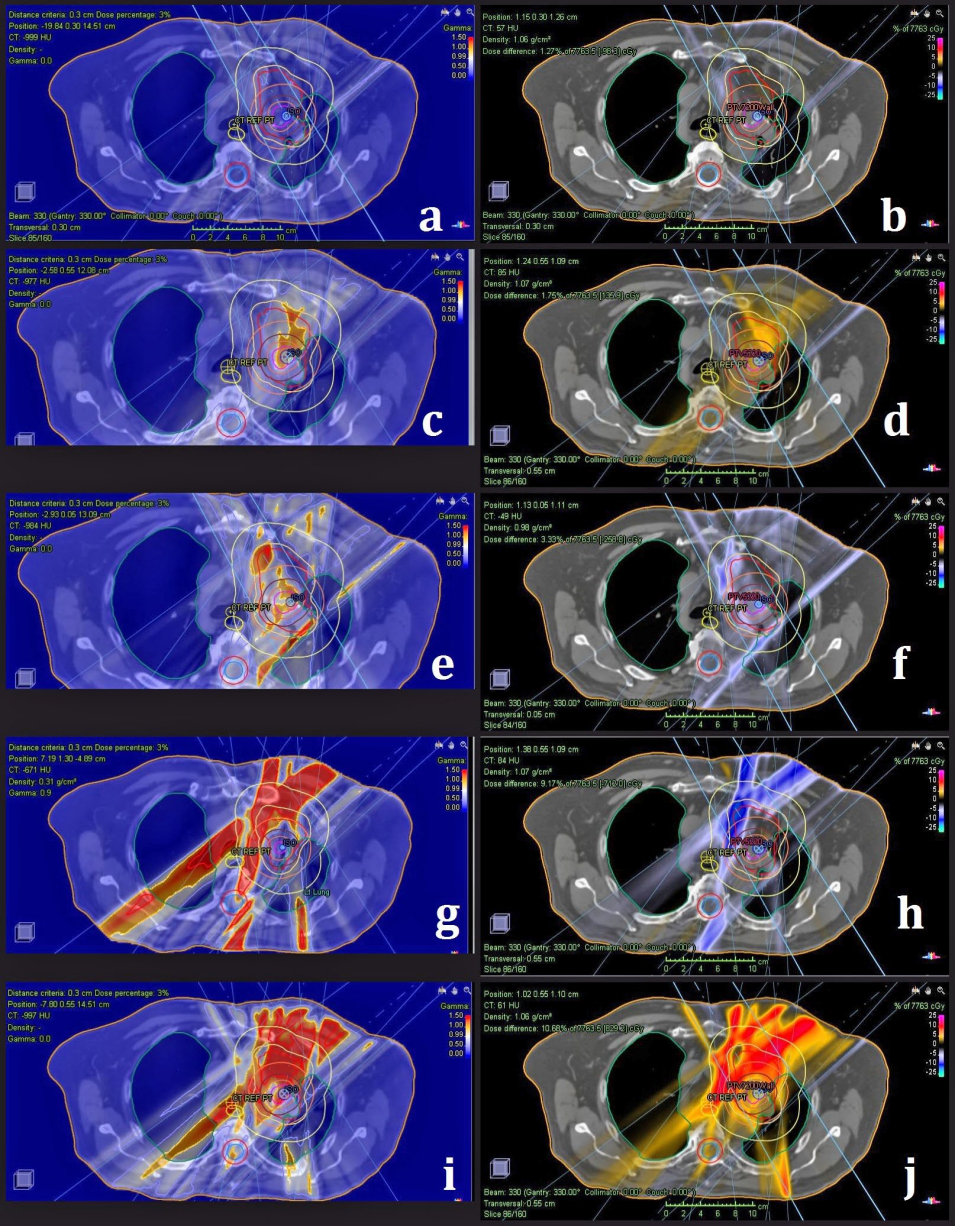
Clinical errors used in this study. Subfigures (a) without introduced error, (c) for 3% MU increase, (e) for 3% MU decrease, (g) for single MLC leaf inserted into the field, (i) for single MLC leaf retracted from the field, show gamma test fail in red overlaid with patient CT for the first thoracic case. Subfigures (b), (d), (f), (h), (j) show TPS computed and COMPASS measured absolute dose difference for the same errors and the same patient.

Additionally, there are certain advantages of 3D QA which are absent in 2D QA. For instance, 3D QA provides the ability to use various statistical parameters to test any treatment plan structure. Dose is computed directly in the patient CT and not in a phantom. With our current setup, only 3D QA can detect gantry angle errors. Another advantage of 3D QA performed with COMPASS system is the ability to overlay absolute dose difference or gamma test results with patient's CT. This is important in cases when some structures or parameters fail QA, while others pass. In such case, one can scroll through the CT slices and investigate where the failure occurs and make much more informed decisions if the failure is so severe that the plan must be reevaluated. This 3D QA feature is also valuable to understand some of the errors introduced in this work that produced mixed results with the other two QA methods. Such errors are MU changed by 3% and single MLC leaf retracted from or placed in the treatment field as seen in Fig. 1. Subfigures (a), (c), (e), (f), and (i) show gamma analysis results overlaid with patient anatomy for those errors. Red color indicates areas where gamma analysis failed the 3%/3 mm DTA criteria. Subfigures (b), (d), (f), and (j) show TPS versus COMPASS measured dose difference overlaid with patient anatomy for those errors.

Our results showed that MLC error results were treatment plan dependent, meaning dependent on the treatment structure size, as well as on the position of the MLC error relative to the MLC modulation pattern. For example, a single central MLC leaf placed in the treatment field is detected by 3D gamma test parameter for both prostate plan target structures (Table 6, prostate structure) and for the first thoracic plan target structure (Table 7, target structure), but barely for the second thoracic plan target structure (Table 7, target structure). This was due the fact that the chosen central MLC leaf was already mostly closed for most of the beam segments.

As mentioned before, detector shift of 5 mm error with 2D gamma test gave mixed results all of which are around the 90th percentile pass criterion used in our institution. A postmeasurement data processing could fix such error and the 2D gamma test would pass, which might be good or bad depending on the error source. If the detector shift was a result of a setup error, being able to fix the error is a good thing. However, if the error was due to all MLC leaves being shifted uniformly to the right or left, the ability to fix such error might lead to false positive QA pass. Another disadvantage of 2D gamma analysis is that it has been previously found to be measurement acquisition plane‐dependent^(6)^ and unable to detect certain errors^(5)^ that would render a given treatment plan clinically unacceptable.

Although current work showed COMPASS system as an excellent tool for 3D QA, we would like to acknowledge some of its drawbacks. One such drawback is significantly longer QA time needed per patient compared to the time needed during single ion chamber or 2D gamma analysis QA. Another drawback is the inability to detect TPS‐ versus measured‐dose differences outside of treatment plan structures included in the QA protocol, such as cold or hot spots. There is also the physical resolution limitation of the MatriXX detector, reduced further by the short SSD.

## V. CONCLUSIONS

In this work we showed the clinical implementation of a 3D quality assurance protocol for prostate and thoracic IMRT. The proposed protocol allows us to perform QA on individual treatment plan structures using multiple statistical parameters. The 3D QA protocol is also sensitive enough to detect most of the introduced clinical errors and is superior to QA performed using 2D gamma analysis or using ion chamber by detecting 14 out of the 18 introduced errors. The two errors not detected by any of the QA methods investigated were MU changed by 1% and 10 MV error. We believe that using 3D QA protocol provides us with in‐depth information about the quality of treatment delivery and allows us to make a fully informed QA decision.

## ACKNOWLEDGMENTS

Contributing author Mr. Bruce Crawford acknowledges travel support from IBA. Gueorgui Gueorguiev acknowledges receiving funding from IBA for work unrelated to this manuscript project.
